# Efficacy and safety of aerosol inhalation of recombinant human interferon α1b (IFNα1b) injection for noninfluenza viral pneumonia, a multicenter, randomized, double-blind, placebo-controlled trial

**DOI:** 10.1186/s12950-020-00249-1

**Published:** 2020-05-14

**Authors:** Rongmeng Jiang, Bing Han, Meihua Song, Bing Xue, Yongxiang Zhang, Yanyan Ding, Jin Chen, Jing Zhu, Jianhua Liu, Qingrong Nie, Xuefeng Han, Xiuhong Jin, Xiaoyin Shan, Weian Guo, Erming Zhang, Zuoqing Zhang, Changhong Zhang, Jie Zhang, Baozeng Wang, Shuwen Dong, Jiandong Li, Xiaoguang Li, Xingwang Li

**Affiliations:** 1 Department of Infectious Disease, Beijing Ditan Hospital, Capital Medical University, No. 8 East Jingshun Street, Chaoyang District, Beijing, 100015 China; 2 Department of Respiratory Medicine, Beijing Chuiyangliu Hospital, Beijing, 100022 China; 3 Department of Respiratory Medicine, People’s Hospital of Beijing Daxing District, Beijing, 102600 China; 4 Department of Respiratory Medicine, Fuxing Hospital, Capital Medical University, Beijing, 100038 China; 5 Department of Respiratory Medicine, Beijing Huairou District Hospital, Beijing, 101400 China; 6 Department of Respiratory Medicine, Liangxiang Hospital of Fangshan District, Beijing, 102401 China; 7 Department of Respiratory Medicine, Beijing Pinggu Hospital, Beijing, 101200 China; 8 Department of Respiratory Medicine, Peking University Shougang Hospital, Beijing, 100144 China; 9 Department of Respiratory Medicine, Beijing Shijingshan Hospital, Beijing, 100043 China; 10 Department of Respiratory Medicine, Beijing Tiantan Hospital, Capital Medical University, Beijing, 100070 China; 11 Department of Infectious Disease, Beijing Tiantan Hospital, Capital Medical University, Beijing, 100070 China; 12 Department of Respiratory Medicine, General Hospital of Beijing Military Region, Beijing, 100700 China; 13 Department of Infectious Disease, Peking University Third Hospital, Beijing, 100191 China

**Keywords:** Noninfluenza viral pneumonia, Recombinant human interferon α1b, Aerosol inhalation, Clinical trial

## Abstract

**Background:**

To investigate the efficacy and safety of aerosol inhalation of recombinant human interferon α1b (IFNα1b) injection for noninfluenza viral pneumonia.

**Methods:**

One hundred sixty-four patients with noninfluenza viral pneumonia were divided into IFNα1b and control groups. The IFNα1b group received routine treatment + aerosol inhalation of recombinant human IFNα1b injection (50 μg × 2 injections, bid). The control group received routine treatment + IFN analog (two injections, bid). Overall response rate (ORR) of five kinds clinical symptoms. Further outcomes were daily average score and the response rate of each of the symptoms above.

**Results:**

A total of 163 patients were included in the full analysis set (FAS) and 151 patients were included in the per-protocol set (PPS). After 7 days of treatment, ORR of clinical symptoms was higher in IFNα1b group than that in control group for both the FAS and PPS. Moreover, after 7 days of treatment, the daily score of three efficacy indexes including expectoration, respiratory rate, and pulmonary rales were improved. The ORRs for expectoration and pulmonary rales were higher in the IFNα1b group than in the control group (*P* < 0.05). There were no significant differences of the ORRs for coughing, chest pain and respiratory rate between the two groups (*P* > 0.05). The incidence of adverse events was 6.5% (*n* = 5) in IFNα1b group and 3.5% (*n* = 3) in control group (*P* > 0.05).

**Conclusion:**

Aerosol inhalation of recombinant human IFNα1b is safe and it can improve the clinical symptoms of noninfluenza viral pneumonia.

## Introduction

Pneumonia is the leading cause of death in children in developing countries and the elderly in developed countries. Among the pathogens which cause community-acquired pneumonia (CAP), viral pneumonia is attracting increasing attention. It occurs in approximately 200 million people each year worldwide, while adults and children each accounting for 50% of cases [1]. In recent years, with the development of molecular assays, respiratory viruses have become the leading CAP pathogens [2]. CAP is the most common infectious disease in the United States and the eighth cause of death [3]. It is reported that the economic burden of CAP in the United States alone was estimated to exceed $17 billion per year [4]. A CAP study in the United States found that the three most common adult pneumonia pathogens were rhinovirus, influenza virus, and *Streptococcus pneumoniae*. Other pathogens include human metapneumovirus, respiratory syncytial virus, parainfluenza virus, coronavirus, and adenovirus [2]. Common viruses include influenza virus (8%), rhinovirus (5.7%), respiratory syncytial virus (2.2%), and coronavirus (3.3%) [5]. In recent years, the mortality of newly developed avian influenza viruses (H7N9, H5N6) and the Middle East respiratory syndrome coronavirus (MERS-CoV) was high. Once 48 h passed the infection, neuraminidase inhibitors were ineffective for influenza virus infections. Currently, no proved effective antiviral treatment is available to treat and prevent MERS. MERS-CoV is highly homologous to severe acute respiratory syndrome (SARS), therefore, the experience gained from SARS treatment, including the use of IFNα, may be helpful in the treatment of MERS-CoV infection. Through in vitro studies, it was found that MERS-CoV was 50 to 100 times more sensitive to IFNα than SARS virus. Therefore, IFNα might be an effective treatment for MERS [6]. IFNα was shown to be effective in patients with chronic active Epstein-Barr virus disease [7, 8]. Sakai et al. reported the application of IFNα to patients with chronic active Epstein-Barr virus disease resulting in unremarkable suppression of lymphocyte proliferation [8]. An open-label trial of systemic IFN improved 28-day survival in patients with ARDS [9].

Interferons (IFNs) are a family of multifunctional cytokines with broad-spectrum antiviral, antiproliferative, and immunomodulatory activities. IFN has broad-spectrum antiviral and immunomodulatory effects and is classified as type I, type II or type III based on different binding receptors [10]. Among them, type I IFN (mainly IIB IFN) plays an important role in the control of viral infections. IFNα establishes an antiviral state by inducing cells to produce antiviral proteins, which prevents viral infection [11]. Aerosol inhalation is one route way for IFN administration, which could not only improve efficacy but also reduce the IFN blood concentration in normal tissues, thereby reducing adverse reactions [12]. In clinical practice, IFNα has been widely used to treat various viral diseases in children in some disease [13, 14], but few studies have been conducted to investigate its effect in the treatment of viral pneumonia in adults. Moreover, there were also no consensus on how to use IFNα in adults with viral pneumonia.

Therefore, we performed a multicenter, randomized controlled study to investigate the efficacy and safety of aerosol inhalation of recombinant human IFNα1b for noninfluenza viral pneumonia and to provide a basis for its rational use and dose in clinical practice.

## Methods

### Study design

This is a multicenter, randomized, double-blind, placebo-controlled trial, which was approved by the Ethics Committee of Beijing Ditan Hospital Capital Medical University. Each subject was informed of the purpose of the study and potential benefits and risks and signed an informed consent before the study. Inclusion Criteria: The inclusion criteria were as follows: 1) men or women at least 14 years old with pneumonia diagnosed according to the *Guidelines for the Diagnosis and Treatment of Adults with Community-Acquired Pneumonia* from the Respiratory Disease Branch of the Chinese Medical Association (2016); 2) clinical diagnosis of viral pneumonia; 3) negative tests for influenza viruses; 4) inpatients, within 5 days of onset; and 5) ability to receive aerosol inhalation.

The exclusion criteria were as follows: 1) unequivocal evidence of *Tuberculosis bacillus*, *Streptococcus pneumoniae*, *Legionella pneumoniae*, *Mycoplasma pneumoniae*, or *Chlamydia* infection; 2) unequivocal evidence of bacterial infection, procalcitonin (PCT) > 1 μg/L; 3) use of antiviral drugs in the week before screening or potential need for another antiviral treatment during the study; 4) subjects who required mechanical ventilation; 5) unstable or active chronic lung disease, diabetes, tumor, or HIV infection; 6) severe liver or kidney dysfunction; 7) participating or participated in another clinical study during the 30 days before study treatment; 8) a history of IFN allergy or other IFN contraindications; 9) pregnant (positive urine or serum pregnancy test) or nursing women.

### Intervention

The patients were randomly divided into IFNα1b group and control group.

The IFNα1b group was given routine treatment (antibiotics and antitussive and expectorant drugs) and aerosol inhalation of recombinant human IFNα1b injection (Beijing Tri-Prime Gene Pharmaceutical Co., Ltd., lot#: 20151206), 50 μg × 2 injections, bid. The control group was given routine treatment and aerosol inhalation of IFN analog (Beijing Tri-Prime Gene Pharmaceutical Co., Ltd.), 2 injections, bid. Standardized compressor nebulizers were used at all sites to administer treatment via the same nebulization process.

### Primary outcome

The primary outcome was the overall response rate (ORR) of five pneumonia-related symptoms, including coughing, expectoration, chest pain, pulmonary rales, and respiratory rate after treatment. The ORR of clinical symptoms (%) = (pretreatment score of clinical symptoms – the score of clinical symptoms after 7 days of treatment) / pretreatment score × 100%. The clinical signs and symptoms were scored according to the *Guiding Principles for Clinical Study of New Chinese Medicines*, 0, no coughing, expectoration, chest pain, or pulmonary rales, and a normal respiratory rate; 1, mild; 2, severe.

### Secondary outcomes

The secondary efficacy measures were daily average score and the response rate of each of the five pneumonia-related symptoms, including coughing, expectoration, chest pain, pulmonary rales, and respiratory rate before and after treatment.

### Safety measures

The measured vital signs included pulse, blood pressure, body temperature, and respiratory rate. The items of physical examination included skin and mucous membranes, lymph nodes, head and neck, chest, abdomen, spine, musculoskeletal system, nervous system. The clinical laboratory tests included complete blood count and serum biochemical examination.

### Statistical analysis

SAS v9.3 was used for statistical analysis. A two-side *p*-value less than 0.05 was considered to be statistically significant. The full analysis set (FAS) was used to analyze primary outcome, and the per-protocol set (PPS) was used to analyze secondary outcomes. Moreover, a superiority test was performed to analyze primary efficacy measures. Fisher’s exact test was used to analyze the overall incidence of adverse events and the incidence of drug-related adverse events.

## Results

### General information

**Fig. 1:**
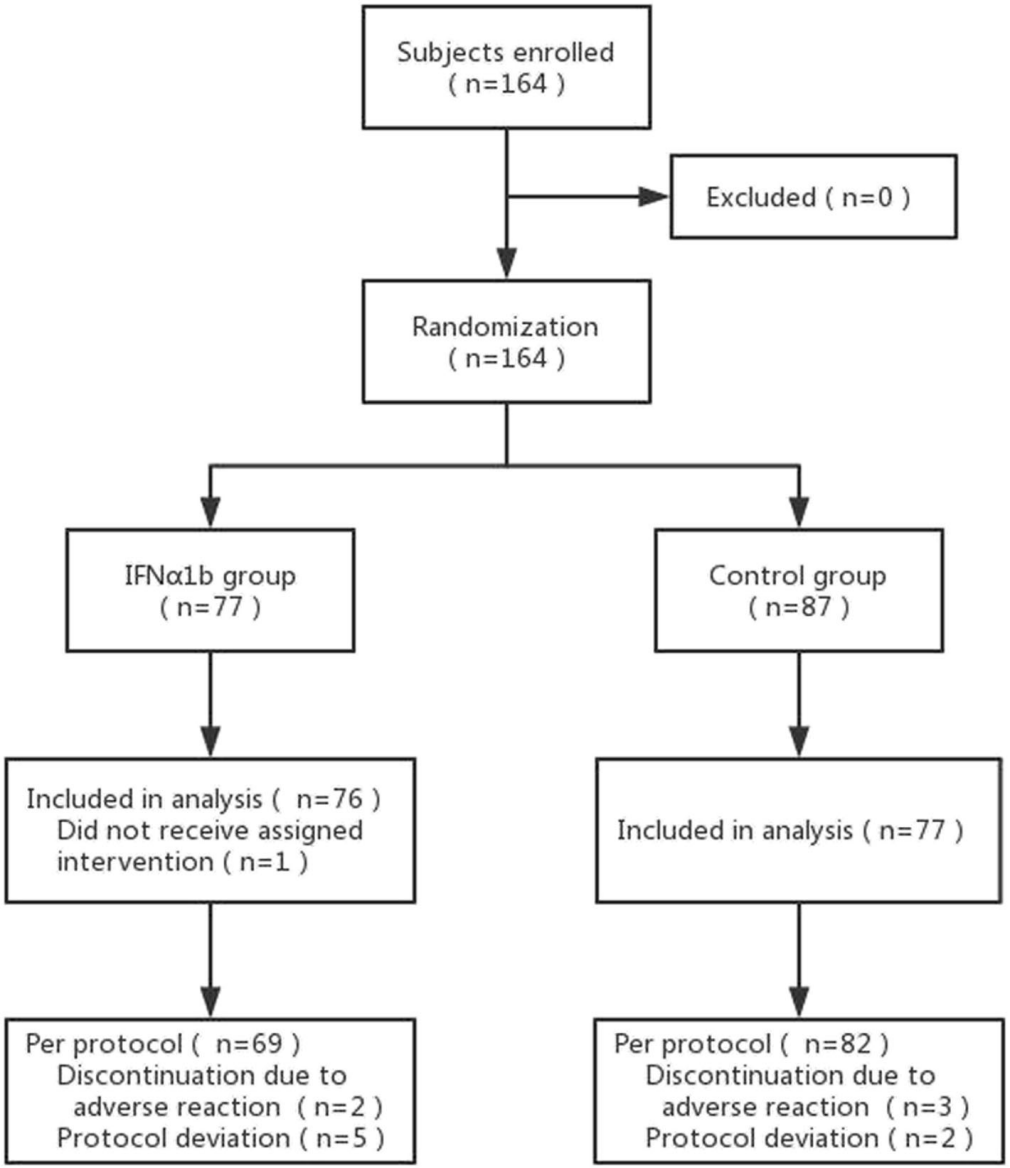
Flow chart of this study

A total of 164 subjects were enrolled in this study. Among them, 163 were included in the FAS, while there were 76 patients in the IFNα1b group and 87 patients in the control group. A total of 151 were included in the PPS, there were 69 patients in the IFNα1b group and 82 patients in the control group. There were no significant differences of age, body mass index (BMI), or time of onset between the two groups (*P* > 0.05). However, there were significant differences of gender composition between the two groups (*P* < 0.05) (Table 1). During subject screening and enrollment, no significant difference was observed in body temperature, respiratory rate, heart rate, pulse, blood pressure, or blood oxygen saturation level (P > 0.05). No subject was transferred to the RICU due to worsening condition and no subject developed secondary bacterial pneumonia after enrollment during the study. The flow chart of subject screening and enrollment is shown in Fig. 1.

**Table 1:** Baseline characteristics of the IFNα1b group and the control group (FAS)

		IFNα1b Group	Control Group	P
		76	87	
Sex	Female	21 (27.6%)	47 (54.0%)	0.001
	Male	55 (72.4%)	40 (46.0%)	
Age		55.230 ± 18.950	51.860 ± 19.528	0.268
BMI		24.306 ± 3.034	23.814 ± 3.179	0.380
Time of onset (days)		5.3 ± 4.2	4.7 ± 2.7	0.239

### Viral pathogens

Identified Viral pathogens in the enrolled subjects were shown in Table 2. Influenza virus, parainfluenza virus, respiratory syncytial virus, rhinovirus, and adenovirus were identified, among which influenza virus was the most common virus (*n* = 16, 9.94%).

**Table 2:** Pathogen Identification

Item	Total	IFNα1b Group	Control Group
Influenza virus^a^	16 (9.94%)	9 (11.84%)	7 (8.24%)
Parainfluenza virus	4 (2.48%)	3 (3.95%)	1 (1.18%)
Respiratory syncytial virus	7 (4.35%)	2 (2.63%)	5 (5.88%)
Rhinovirus	7 (4.35%)	3 (3.95%)	4 (4.71%)
Adenovirus	7 (4.35%)	5 (6.58%)	2 (2.35%)
EB virus	16 (9.94%)	7 (9.21%)	9 (10.59%)
Herpes virus	14 (8.64%)	8 (10.53%)	6 (7.06%)
Coronavirus	2 (1.24%)	1 (1.32%)	1 (1.18%)
Human metapneumovirus	1 (0.62%)	0 (0.00%)	1 (1.18%)
*Streptococcus pneumoniae*	2 (1.24%)	2 (2.63%)	0 (0.00%)
Mycoplasma	10 (6.21%)	4 (5.26%)	6 (7.06%)
Chlamydia	2 (1.24%)	1 (1.32%)	1 (1.18%)
Moraxella catarrhalis	2 (1.24%)	0 (0.00%)	2 (2.35%)
Simple virus infection	49 (30.43%)	21 (27.63%)	28 (32.94%)
Single virus infection	30 (18.63%)	9 (11.84%)	21 (24.71%)
Two-virus infection	9 (5.59%)	7 (9.21%)	2 (2.35%)
Multivirus infection	10 (6.21%)	5 (6.58%)	5 (5.88%)
Mycoplasma + virus	5 (3.11%)	2 (2.63%)	3 (3.53%)
Chlamydia + virus	2 (1.24%)	1 (1.32%)	1 (1.18%)

^a^represents negative results from a rapid influenza antigen test during screening and positive results from an influenza virus nucleic acid test at the central laboratory. No anti-influenza drugs were given during clinical treatment.

### ORR of clinical symptoms after 7 days of treatment

For FAS analysis, the ORR of clinical symptoms was 76.87 ± 25.15% in the IFNα1b group and 65.51 ± 35.59% in the control group after 7 days of treatment. For PPS analysis, the ORRs were 77.16 ± 24.19% and 66.26 ± 35.65%, respectively. In both FAS and PPS, the ORR was significantly higher in the IFNα1b group compared with that in the control group (*P* < 0.05) (Table 3). Covariance analysis showed there was no significant correlation between gender composition and ORRs (*P* > 0.05) (Table 4).

**Table 3:** ORRs of clinical symptoms in the IFNα1b group and the control group after days of treatment (%)

Group	FAS	ORR	PPS	ORR
	n		n	
Control group	87	65.51 ± 35.59	82	66.26 ± 35.65
IFNα1b group	76	76.87 ± 25.15	69	77.16 ± 24.19
P	0.037		0.033

**Table 4:** Covariance analysis of ORRs of clinical symptoms between the IFNα1b group and the control group after 7 days of treatment

	FAS	P	PPS	P
	Statistics F		Statistics F	
Covariate effect	0.557	0.457	0.317	0.574
Sex comparison	0.929	0.337	1.493	0.224
Between-group comparison	4.781	0.030	3.649	0.048
Sex and Group	0.006	0.941	0.043	0.836

Subjects with a positive influenza virus test were excluded from the original FAS to establish a new FAS. Analysis of the new FAS showed that the ORRs of clinical symptoms after 7 days of treatment were consistent with the original FAS results (Table 5).

**Table 5:** Description and comparison of ORRs of clinical symptoms between the IFNα1b group and the control group after 7 days of treatment

Group	n	ORR
Control group	80	66.66 ± 36.29
IFNα1b group	67	77.93 ± 22.35
P		0.044

### Seven-day daily score for each symptom

**Fig. 2:**
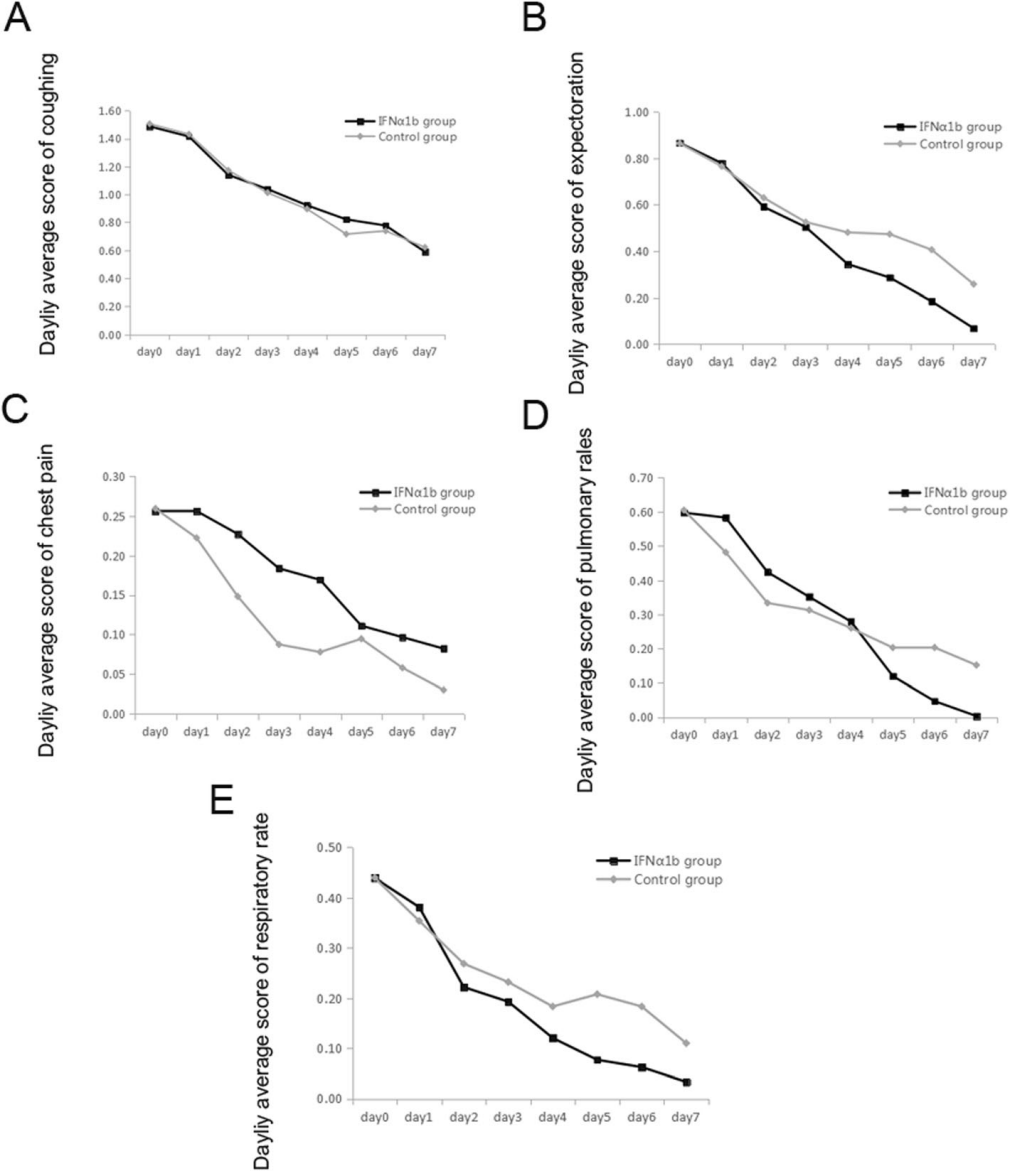
Daily Scores of Coughing (**a**), Expectoration (**b**), Chest Pain (**c**), Pulmonary Rales (**d**) and Respiratory Rate (**e**) in the IFNα1b Group and the Control Group

During the treatment, the scores of three efficacy measures (expectoration, respiratory rate, and pulmonary rales) decreased rapidly, especially the expectoration and respiratory rate (Fig. 2). On days 2 and 3 of the treatment, the IFNα1b group showed the most significant improvement in expectoration and respiratory rate. On day 4, the IFNα1b group showed the most significant improvement in pulmonary rales. At the end of treatment, the ORRs of expectoration, respiratory rate, and pulmonary rales remained higher in the IFNα1b group than that in the control group.

### Response rate of each of the five symptoms before and after treatment

The response rates of expectoration and pulmonary rales were significantly higher in the IFNα1b group than that in the control group (*P* < 0.05) (Table 6). No significant differences of other three symptoms, including coughing, chest pain, and respiratory rate, were observed between the two groups (*P* > 0.05).

**Table 6:** Response rate of each of the five symptoms before and after Treatment

Symptoms	IFNα1b Group	Control Group	W	P
Coughing	0.61 ± 0.40	0.52 ± 0.40	3706.5	0.156
Expectoration	0.70 ± 0.44	0.53 ± 0.49	3897.5	0.024
Chest pain	0.16 ± 0.37	0.20 ± 0.40	3182	0.535
Pulmonary rales	0.49 ± 0.49	0.34 ± 0.46	3799.5	0.042
Respiratory rate	0.43 ± 0.50	0.32 ± 0.47	3677.5	0.141

### Survival rate and readmission within 90 days of discharge

Phone follow-ups showed no deaths within 90 days of discharge. Five subjects in the IFNα1b group and four subjects in the control group were readmitted into a general ward (non-ICU) within 90 days of discharge due to complaints other than noninfluenza viral pneumonia. Fisher’s exact probability test showed no significant between-group difference (*P* > 0.05) for both the FAS and PPS (Table 7).

**Table 7:** Readmissions within 90 Days of Discharge

		FAS		PPS
Item	IFN α1b Group	Control Group	IFN α1b Group	Control Group
Readmission within 90 days of discharge	5 (6.6%)	4 (4.6%)	5 (7.2%)	4 (4.9%)
No readmission within 90 days of discharge	71 (93.4%)	83 (95.4%)	64 (92.8%)	78 (95.1%)
Total (Missing)	76 (0)	87 (0)	69 (0)	82 (0)
Statistics	Fisher’s exact probability test		Fisher’s exact probability test	
P	0.735		0.733	

### Antibiotic use

The rate of antibiotic use was 93.42% in the IFNα1b group and 90.80% in the control group (*P* > 0.05) (Table 8). The duration of antibiotic use was 7.84 ± 3.50 days in the IFNα1b group and 7.99 ± 3.78 days in the control group (*P* > 0.05) (Table 9). A quantitative virus test was performed before treatment, on days 3 and 7 of treatment in the IFNα1b group. The virus nucleic acid load was significantly decreased on day 7 of treatment relative to that before treatment.

**Table 8:** Rate of antibiotic Use in the IFNα1b group and the control group (%)

	FAS		PPS	
	IFNα1b Group	Control Group	IFNα1b Group	Control Group
Use of Item antibiotics	5 (6.6%)	8 (9.2%)	5 (7.2%)	7 (8.5%)
Use of antibiotics	71 (93.4%)	79 (90.8%)	64 (92.8%)	75 (91.5%)
P	0.745		1.000

**Table 9:** Duration of antibiotic use in the IFNα1b group and the control group

	FAS		PPS	
Item	IFNα1b Group	Control Group	IFNα1b Group	Control Group
n (Missing)	140 (24)	138 (22)	122 (21)	131 (20)
Mean (SD)	7.84 (3.50)	7.99 (3.78)	7.83 (3.25)	7.95 (3.83)
Median	8	8	8	8
Q1, Q3	5.0, 9.3	5.8, 10.3	6.0, 9.0	5.0, 10.5
Min, Max	1,16	1,16	1,16	1,16
95% CI	7.20, 8.49	7.30, 8.69	7.19, 8.47	7.24,8.67
Statistics	Wilcoxon rank sum test	6523	Wilcoxon rank sum test	5416
P	0.688		0.670	

### Safety evaluation

No significant differences of the vital signs (pulse, blood pressure, body temperature, respiratory rate) of the 164 subjects were observed between the two groups (*P* > 0.05). Physical examinations, including examinations of the skin and mucous membranes, lymph nodes, head and neck, chest, abdomen, spine, musculoskeletal system, and nervous system, revealed no clinically significant abnormal findings. In urine analysis, stool tests, electrocardiography, and blood oxygen saturation level measurements, few subjects showed clinically significant abnormalities, and no significant between-group differences were found (*P* > 0.05). At the end of the treatment, the frequency of abnormal blood and biochemical tests was 176 cases in the IFNα1b group and 158 cases in the control group (*P* > 0.05).

The statistics of adverse events were shown in Table 10. The incidence of adverse events was 6.5% in the IFN α1b group and 3.5% in the control group. During treatment, two subjects in the IFNα1b group had a rash but continued to receive IFN treatment. Their rash disappeared after antiallergic treatment. One subject in the IFNα1b group had a sore throat and dry throat, and the symptoms resolved after IFN discontinuation. Two subjects in the IFNα1b group and one subject in the control group had a low white blood cell (WBC) count, which was resolved after IFN discontinuation. Moreover, one patient in the control group was pregnant and thus discontinued treatment.

**Table 10:** Adverse events during the study

		Rash	Sore Throat, Dry Throat	Low WBC	Elevated transaminase	Cessation of menstruation (Pregnancy)	Total
Control group (*n* =87)	n	0	0	2	0	1	3
	Severity	—	—	Moderate	—	Severe	
	Relation to the investigational drug	—	—	Unrelated	—	Unrelated	
IFNα1b group (*n* =77)	n	2	1	1	1	0	5
	Severity	Mild	Mild	Mild	Moderate	—	
	Relation to the investigational drug	Possible	Possible	Unrelated	Unrelated	—	

## Discussion

The current study investigated the efficacy and safety of aerosol inhalation of recombinant human interferon α1b (IFNα1b) injection for noninfluenza viral pneumonia. It was found that aerosol inhalation of recombinant human IFNα1b could improve the ORRs of the clinical symptoms with our additional adverse events in noninfluenza viral pneumonia.

This study found that the ORRs of primary efficacy measures, including coughing, expectoration, pulmonary rales, respiratory rate, and chest pain were higher in the IFNα1b group than that in the control group, which received routine symptomatic treatment alone, suggesting that aerosol inhalation of recombinant human INFα1b effectively improves the overall response rate of the disease. IFNα is a recognized immunomodulatory therapy to suppress viral replication by inhibiting basal transcription processes.

Because of its antiviral effects, IFNα has been used in trials in combination with other antiviral agents to prevent and treat emerging and reemerging virus infections for which no approved drugs are available [15–18]. However, results from these trials have yielded inconsistent results. In addition, other studies indicated that IFNα has pathogenic effects during acute and chronic infections [19–23]. Together, these findings suggest that the relationship between virus replication and or related pathogenesis and the kinetics of IFN expression, whether endogenous or after exogenous administration, contributed to the variability of outcomes. IFNα therapy has been used to treat patients with severe respiratory disease caused by CoVs, with similarly inconsistent outcomes [24]. In particular, IFNα treatment of patients with MERS failed to improve survival [15, 25, 26]. For example, in one study, IFN treatment prolonged survival when assessed at 14 days, but not at 28 days [15]. Previous studies have demonstrated a critical role for MDA5 in sensing CoV RNA and thereby initiating the IFN response [27]. The majority of these studies used macrophages infected with a murine CoV, mouse hepatitis virus, which is macrophage tropic [28, 29]. Since human CoVs predominantly infect airway and alveolar epithelial cells and PRR expression is cell-type specific, we examined the PRRs necessary for IFN production specifically in airway and alveolar epithelial cells using mice transduced with Ad5-hDPP4 and then infected with the human EMC/2012 strain of MERS-CoV. Ad5 predominantly infects epithelial cells and not myeloid cells [30]. Although there was no significant difference of chest pain between the two groups. It could still be found that in early 4 days of the treatment, the average score of chest pain seemed to be lower than IFNα1b group. It was reported that the treatment of IFNα1b could lead to some adverse events, including pulmonary effusion cardiac insufficiency [31]. Therefore, the mechanism and reason of the early chest pain still needed to be further investigated.

Prophylactic or early therapeutic administration of IFNs during MERS-CoV infection in rhesus macaques provided significant protection [32]. However, studies in humans failed to conclusively establish the beneficial effects of rIFN therapy [25, 26, 33], possibly because of delayed administration relative to peak virus titers. In the early stage of treatment (days 2 to 3), expectoration, respiratory rate, and pulmonary rales were improved. Virus replication occurs in the early stage, therefore, early treatment may better inhibit virus replication. This study shows that IFN is more effective in patients diagnosed in the early stage, indicating that early treatment is advantageous for disease control and remission, although late treatment is also effective.

When used in the systemic therapy, IFNs are mostly administered by an intramuscular injection. The most frequent adverse effects are flu-like symptoms, especially increased body temperature. In current study, no significant difference of the adverse events was observed between the IFNα1b group and the control group, suggesting that aerosol inhalation of recombinant human IFNα1b injection was safe and well tolerated.

There were also some limitations in this study. First the analysis of viral pathogens could be uncertain, and not all cases have positive pathogen results. Second, the specimen was collected from the upper respiratory tract, which might not be the same pathogen as the lower respiratory tract.

## Conclusion

In conclusion, aerosol inhalation of recombinant human IFNα1b combined with routine treatment is safe and well tolerated in the treatment of noninfluenza viral pneumonia. It significantly improves the ORRs of clinical symptoms and accelerates recovery compared with routine treatment.

## Data Availability

N/A
